# Supplementary material for: Hyperconfined bio-inspired Polymers in Integrative Flow-Through Systems for Highly Selective Removal of Heavy Metal Ions
Source: Nat Commun. 2024 Jul 11;15:5824. doi: 10.1038/s41467-024-49869-8 (PMC11239941; doi:10.1038/s41467-024-49869-8)
Supplement: Supplementary file 1 — Supplementary Information [file 41467_2024_49869_MOESM1_ESM.pdf]

Supplementary Information

Hyperconfined Bio-Inspired Polymers in Integrative Flow-Through Systems for Highly Selective Removal of Heavy Metal Ions

Masaki Nakahata[1,2]*, Ai Sumiya[2], Yuka Ikemoto[3], Takashi Nakamura[4], Anastasia Dudin[5], Julius Schwieger[5], Akihisa Yamamoto[6,†], Shinji Sakai[2], Stefan Kaufmann[5], and Motomu Tanaka[5,6]*

[1]Department of Macromolecular Science, Graduate School of Science, Osaka University, Osaka, 560-0043, Japan

[2]Department of Materials Engineering Science, Graduate School of Engineering Science, Osaka University, Osaka, 560-8531, Japan

[3]Japan Synchrotron Radiation Research Institute (JASRI) SPring-8, Hyogo, 679-5198, Japan

[4]Institute of Pure and Applied Sciences and Tsukuba Research Center for Energy Materials Science (TREMS), University of Tsukuba, Ibaraki, 305-8571, Japan

[5]Physical Chemistry of Biosystems, Institute of Physical Chemistry, Heidelberg University, Heidelberg, 69120, Germany

[6]Center for Integrative Medicine and Physics, Institute for Advanced Study, Kyoto University, Kyoto, 606-8501, Japan

[†]Present address: Interdisciplinary Theoretical and Mathematical Sciences Program (iTHEMS), RIKEN, Saitama, 351-0198, Japan

Preparation of S-trityl-cysteine Acrylamide (*S*-Tri-Cys-AAm).

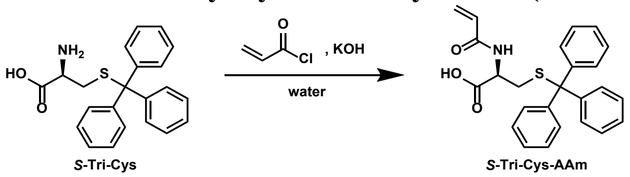

Supplementary Fig. 1. Synthetic scheme for *S*-Tri-Cys-AAm.

S-Tri-Cys-AAm was prepared according to the procedure by Podasca et al.[1] with a slight modification. *S*-Trityl-L-cysteine (2.00 g, 5.5 mmol) and KOH (0.622 g, 11 mmol) were dissolved in water (56 mL) in a water bath at room temperature. Acryloyl chloride (0.60 mL, 7.9 mmol) was dropwise added with stirring. The reaction mixture was stirred at room temperature for another 2 h and then acidified to pH~2 using conc. HCl. The crude product as a solid was collected by filtration and dried under reduced pressure at room temperature. The product was purified with silica gel column chromatography (chloroform-methanol). After evaporation of the solvent, *S*-Tri-Cys-AAm was recovered as a pale-yellow powder (1.71 g, 4.1 mmol, 74%). ^{1}H NMR spectrum (Supplementary Fig. 2b) showed good agreement with previous report.[1] HRMS (ESI) *m*/*z*: $[M + Na]^+$ calcd for $C_{25}H_{23}NNaO_3S$, 440.1296; found, 440.1290.

Synthesis and Characterization of pAA–Cys5.

pAA–Cys5 was synthesized by reversible addition−fragmentation chain-transfer (RAFT) radical polymerization of *S*-Tri-Cys-AAm and acrylic acid (AA) using azobisisobutyronitrile (AIBN) as an initiator and 2-(dodecylthiocarbonothioylthio)-2-methylpropionic acid (DDMAT) as a chain transfer agent, followed by deprotection of trityl group with trifluoroacetic acid (TFA). Briefly, *S*-Tri-Cys-AAm (0.05 mmol), AA (0.95 mmol), AIBN (0.01 mmol), and DDMAT (0.01 mmol) were dissolved in DMSO (1 mL) dried with molecular sieves 4A. The solution was purged with nitrogen gas for 1 h, sealed, and heated in an oil bath thermostated at 65 °C overnight. The solution was poured into acetone (10 mL) with stirring. The resultant viscous concentrated phase was collected with centrifugation (3,500 rpm, 5 min). After

removing the supernatant by decantation, TFA (1 mL) was added and stirred for overnight. The resultant solution was poured into diethyl ether (10 mL). The resultant precipitate was washed with diethyl ether (10 mL) three times, and dried under reduced pressure at room temperature.

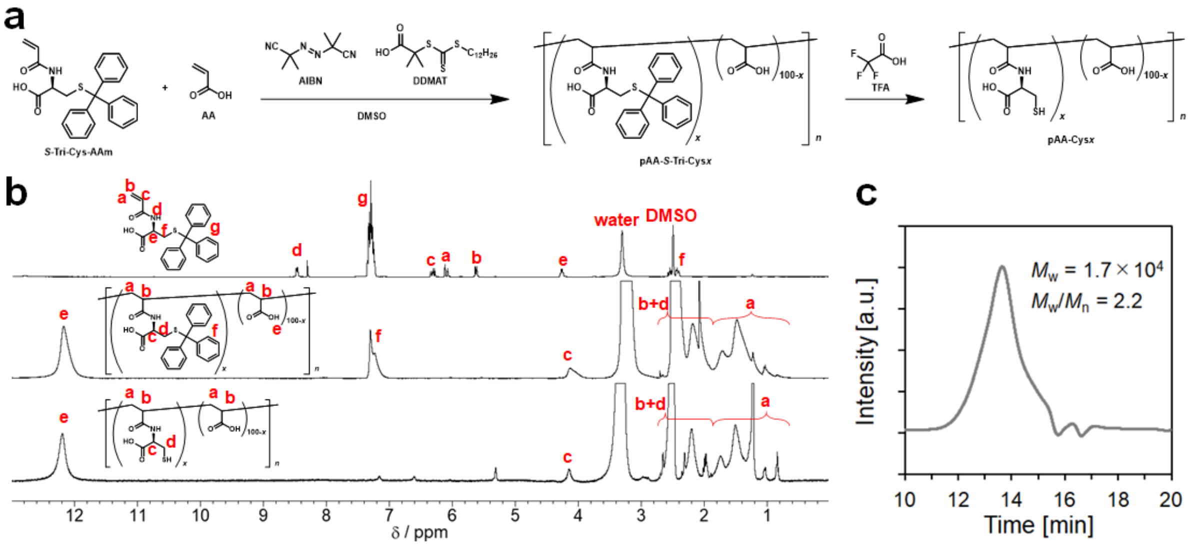

Supplementary Fig. 2. (a) Representative synthetic scheme of pAA–Cys*x*. (b) ^{1}H NMR spectra for *S*-Tri-Cys-AAm, pAA–*S*-Tri-Cys5, and pAA–Cys5 (400 MHz, DMSO-*d*$_6$, 30 °C). The ratio of integral values for a and f peaks in the spectrum of pAA–*S*-Tri-Cys5 are 200 : 73.1, corresponding to 4.9 mol% of *S*-Tri-Cys-AAm units in the resultant polymer. (c) GPC chart for pAA–Cys5 (10 mM Tris-HCl buffer (pH 7.4) + 100 mM NaCl).

ITC Measurements for pAA.

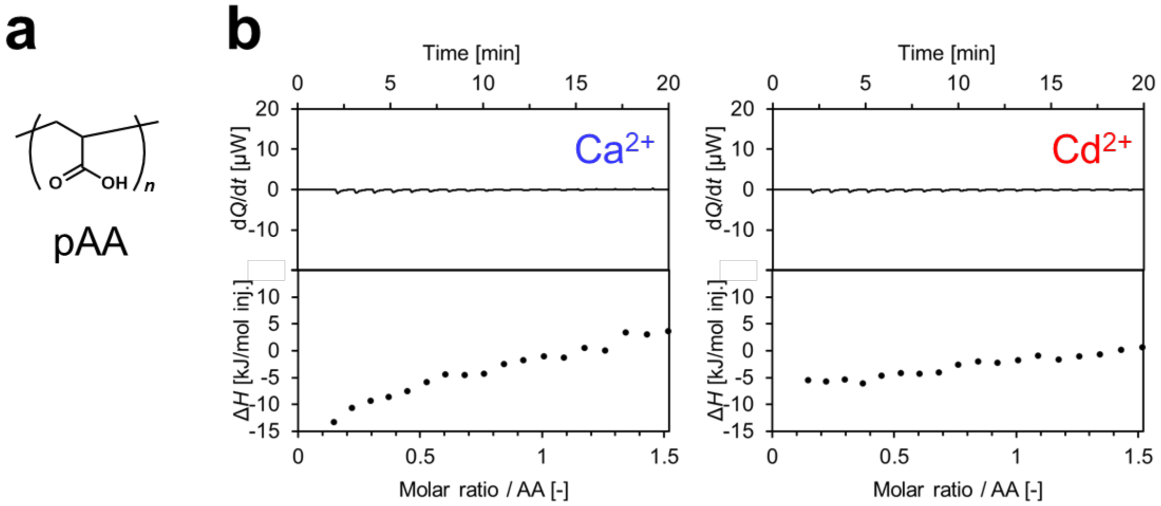

Supplementary Fig. 3. (a) Chemical structure of pAA. (b) Plots of additional thermal power (dQ/dt) and the enthalpy (ΔH) versus the molar ratio of the acrylic acid unit, obtained by the titration of pAA with $CaCl_2$ and $CdCl_2$, respectively.

ITC Measurements.

Supllementary Table 1. Comparison of ITC results with other studies.

**Reference**	**Material**	**Method**	**Conditions**	K_D **/ M**
Chekmeneve et al.[2]	Glutathione	ITC	0.02 M Tris-HCl buffer + 0.1 M NaCl (pH 7.4)	2.0×10^{-5}
Chekmeneve et al.[2]	(γGlu-Cys)$_4$-Gly	ITC	0.02 M Tris-HCl buffer + 0.1 M NaCl (pH 7.4)	4.2×10^{-6}
Cheng et al.[3]	(αGlu-Cys)$_4$-Gly	UV-Vis	0.1 M Tris-HCl buffer (pH 7.4)	3.2×10^{-7}
Visvanathan et al.[4]	oligo(L-Glu-*co*-L-Cys)	UV-Vis	0.1 M Tris-HCl buffer (pH 7.4)	8.6×10^{-4}
This work	pAA–Cys5	ITC	0.01 M Tris-HCl buffer (pH 7.4)	2.1×10^{-9}

IR Measurements.

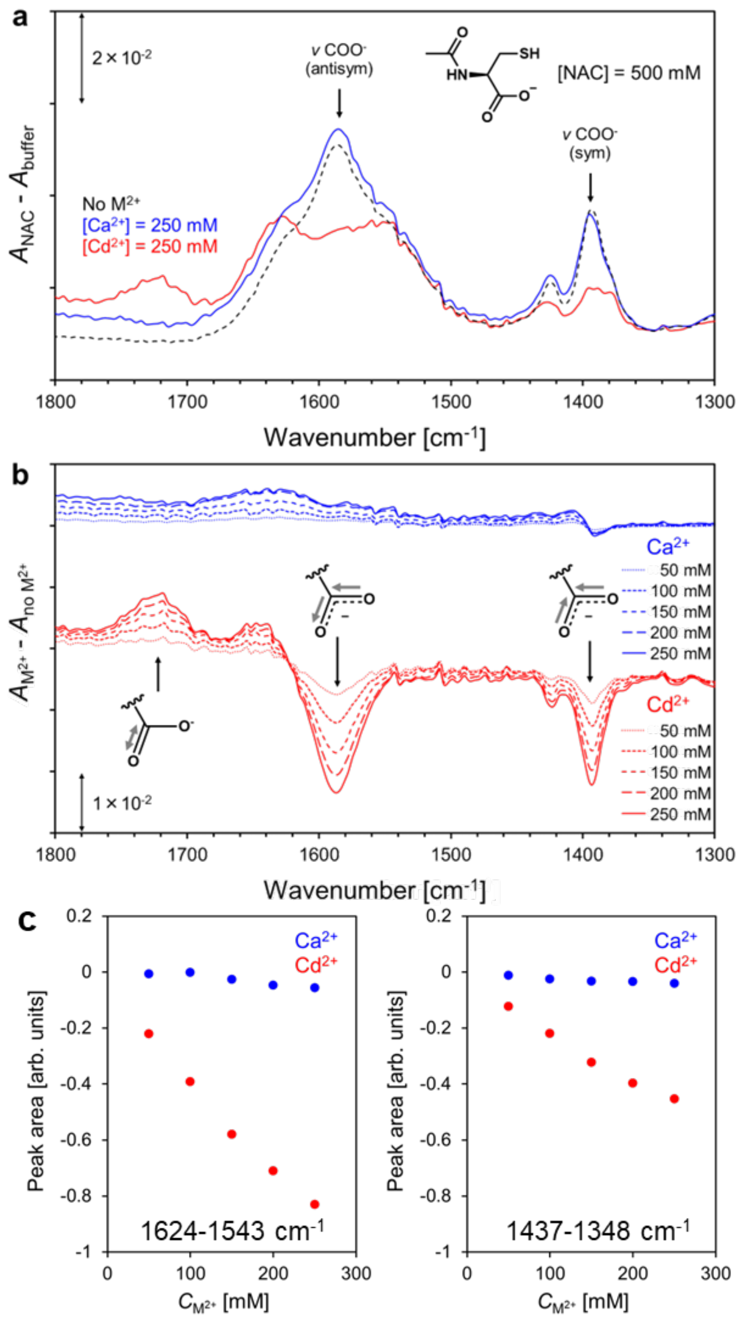

Supplementary Fig. 4. (a) ATR-IR spectra obtained by subtracting the spectrum for a buffer from that for NAC (500 mM) in the absence and presence of Ca^{2+} or Cd^{2+} (250 mM). (b) ATR-IR spectra obtained by subtracting the spectrum for NAC (500 mM) from that for NAC (500 mM) in the presence of Ca^{2+} or Cd^{2+} (50, 100, 150, 200, 250 mM). (c) Peak area (1624 – 1543 and 1437 – 1348 cm^{-1}) plotted against the concentration of Ca^{2+} or Cd^{2+} for (b).

NMR Measurements.

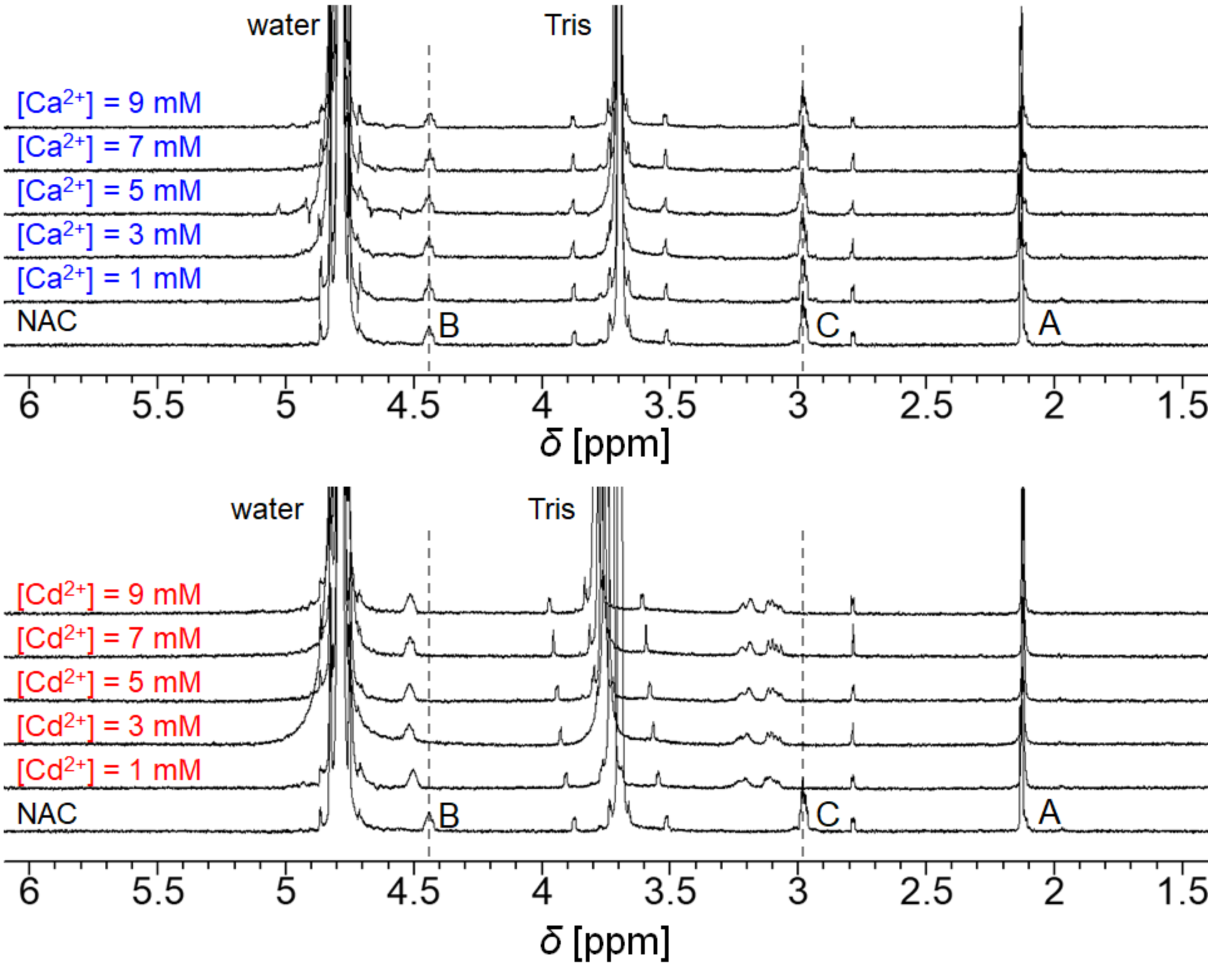

Supplementary Fig. 5. ^{1}H NMR spectra of NAC (1 mM) in the absence and presence of Ca^{2+} or Cd^{2+} (1, 3, 5, 7, 9 mM) in Tris-DCl buffer (10 mM, pD 7.4).

Synthesis and Characterization of pHPMA–Cys5.

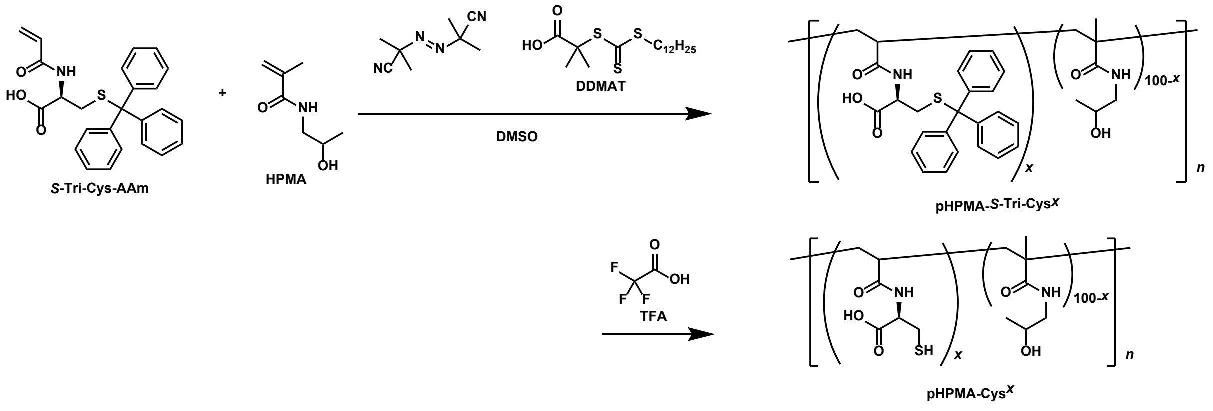

Supplementary Fig. 6. Synthetic scheme for pHPMA–Cys5.

pHPMA–Cys5 was synthesized following almost the same protocol as that of pAA–Cys5 with a slight modification: hydroxypropylmethacrylamide (HPMA) was used instead of AA.

The M_w and M_w/M_n values of pHPMA-Cys5 were determined by GPC (10 mM Tris-HCl buffer (pH 7.4) + 100 mM NaCl) to be 1.9 × 10^4 and 3.4, respectively.

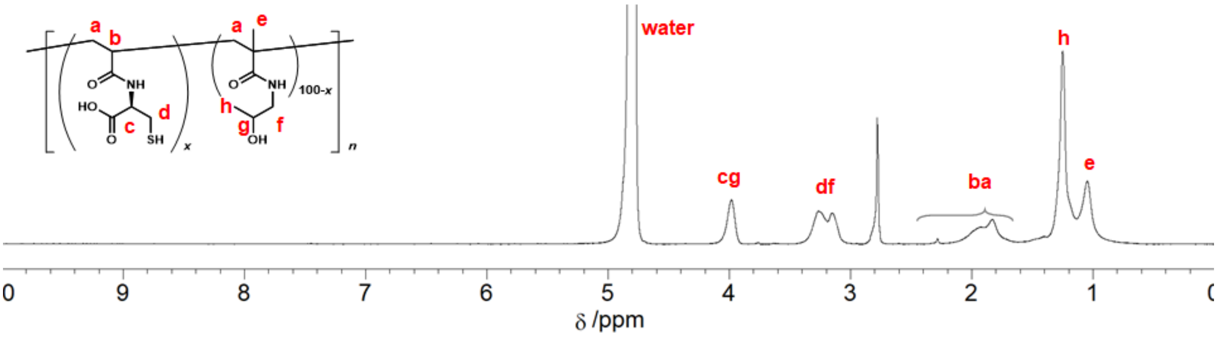

Supplementary Fig. 7. ^{1}H NMR spectrum for pHPMA–Cys5 (400 MHz, D_2O, 30 °C).

Synthesis and Characterization of pPEGMA–Cys5.

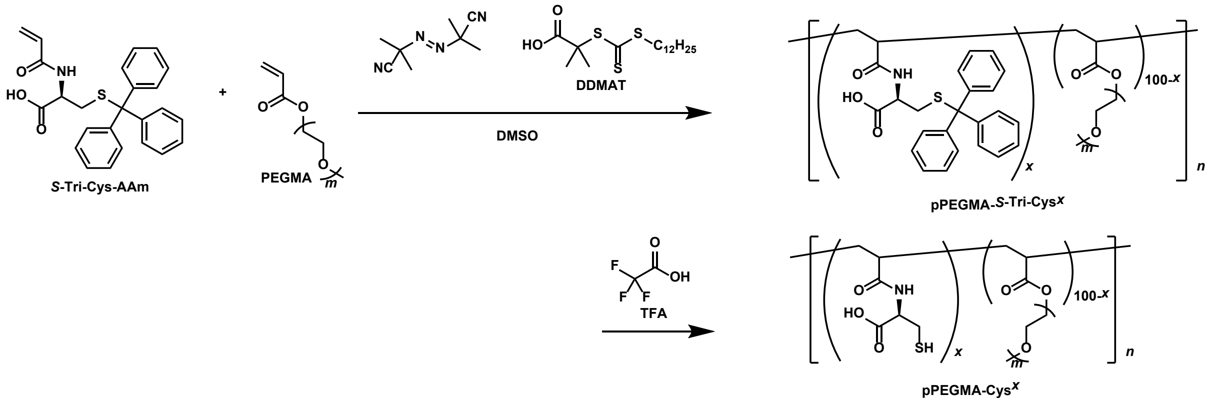

Supplementary Fig. 8. Synthetic scheme for pPEGMA–Cys5.

pPEGMA–Cys5 was synthesized following almost the same protocol as that of pAA–Cys5 with a slight modification: poly(ethylene glycol methyl acrylate) (PEGMA) was used instead of AA; a mixed solvent of diethyl ether and hexane (1/1, v/v) was used instead of diethyl ether. The M_w and M_w/M_n values of pPEGMA–Cys5 were determined by GPC (10 mM Tris-HCl buffer (pH 7.4) + 100 mM NaCl) to be 3.8 × 10^4 and 2.7, respectively.

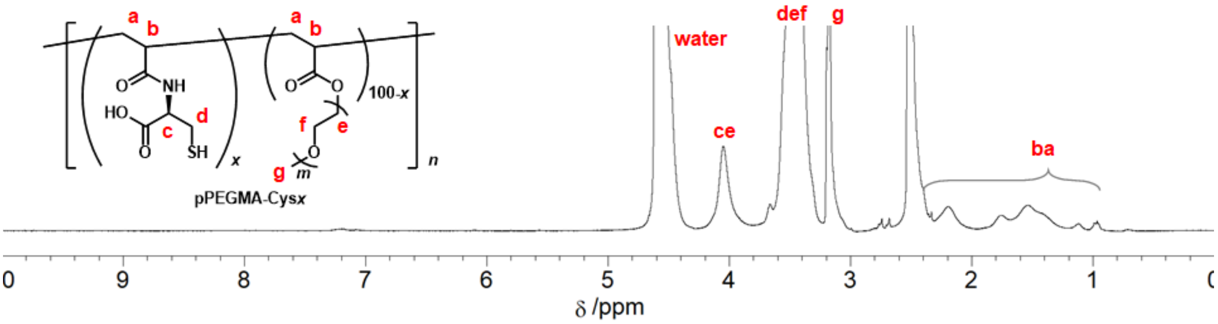

Supplementary Fig. 9. ^{1}H NMR spectrum for pPEGMA–Cys5 (400 MHz, D_2O, 30 °C).

ITC Measurements for pHPMA–Cys5 and pPEGMA–Cys5.

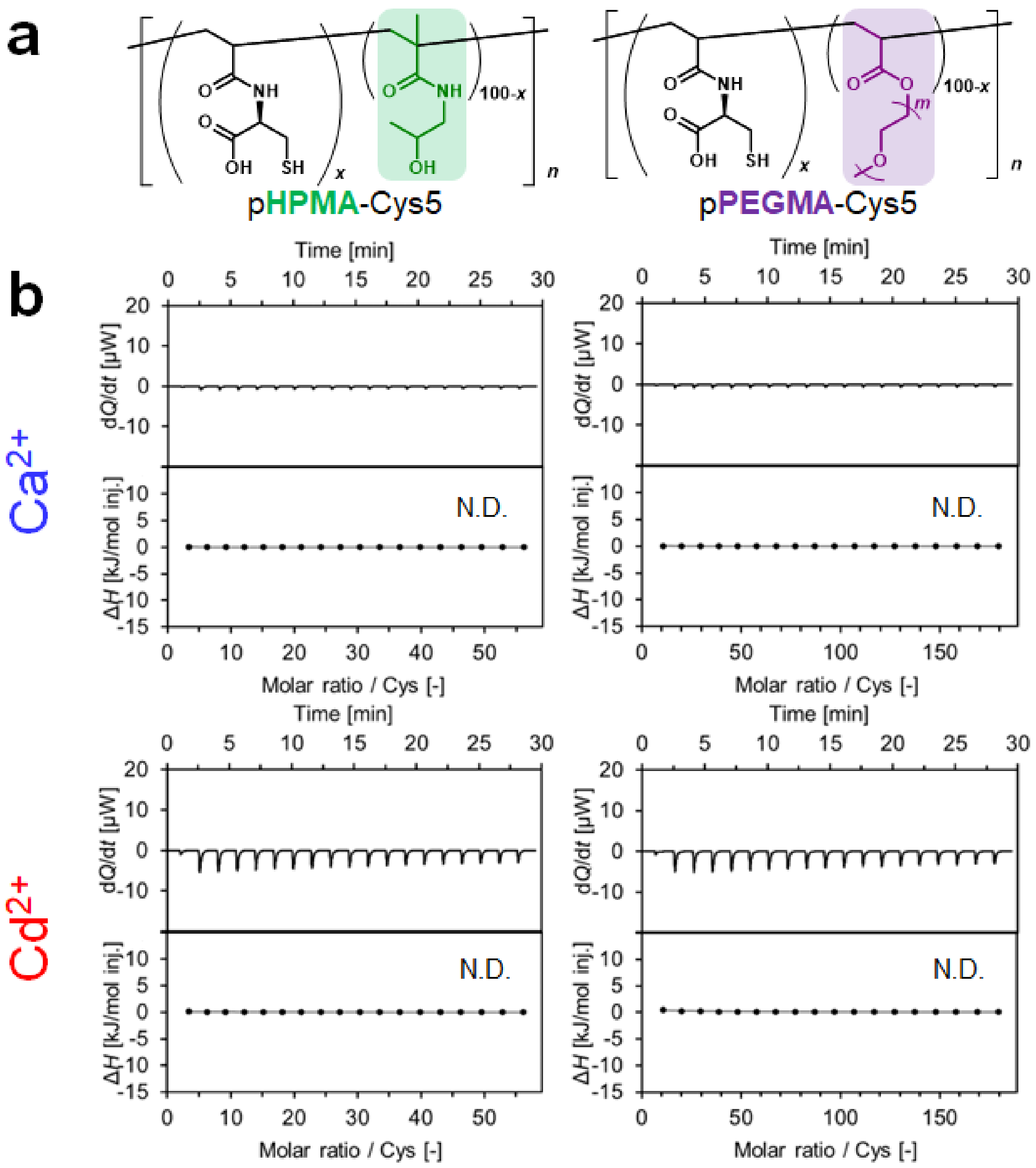

Supplementary Fig. 10. (a) Chemical structures of pHPMA–Cys5 and pPEGMA–Cys5. (b) Plots of additional thermal power (d*Q*/d*t*) and the enthalpy (Δ*H*) versus the molar ratio of the cysteine side chain, obtained by the titration of pHPMA–Cys5 and pPEGMA–Cys5 with $CaCl_2$ and $CdCl_2$, respectively.

Ultrafiltration experiments for ion mixtures.

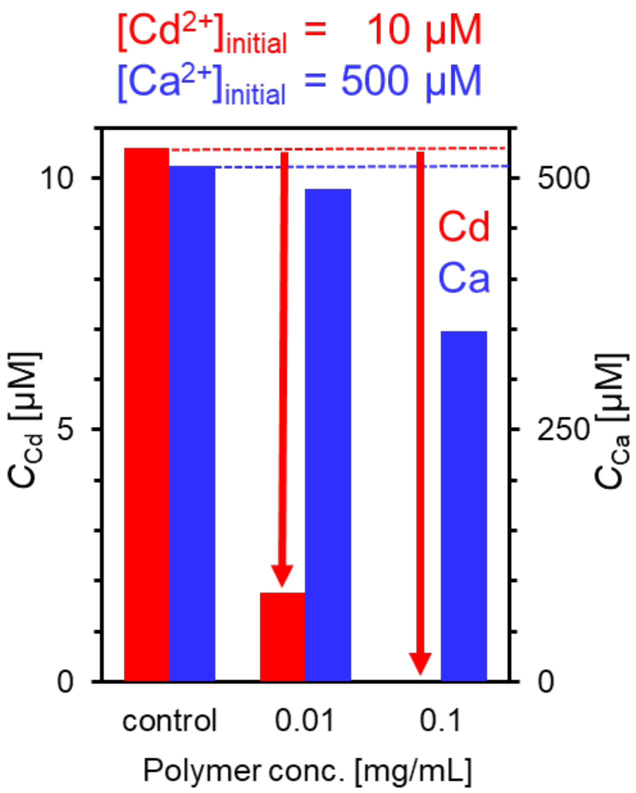

Supplementary Fig. 11. Concentrations of Ca^{2+} and Cd^{2+} in the flow-through after mixing Cd and Ca ($[Cd^{2+}]_{initial}$ = 10 µM, $[Ca^{2+}]_{initial}$ = 500 µM) without or with 0.01 or 0.1 mg mL^{-1} of pAA–Cys5.

ITC Measurements for pAA–Cys5 with abundant mono- and divalent metal ions in ground water.

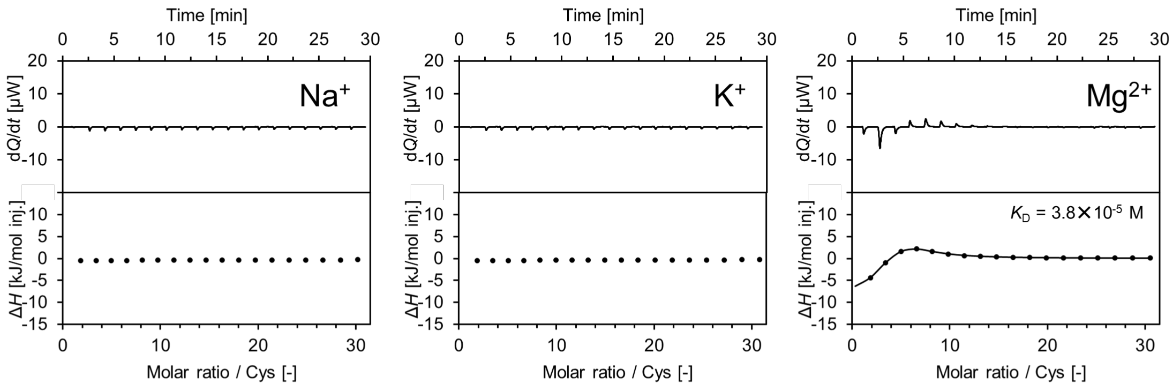

Supplementary Fig. 12. Plots of additional thermal power (d*Q*/d*t*) and enthalpy (ΔH) versus the molar ratio of the cysteine side chain by titrating pAA–Cys5 with NaCl, KCl, and $MgCl_2$, respectively. For $MgCl_2$, the best-fit curves for ITC data using a two-site model are shown (solid lines).

Synthesis and Characterization of end-functionalized pAA–Cys5.

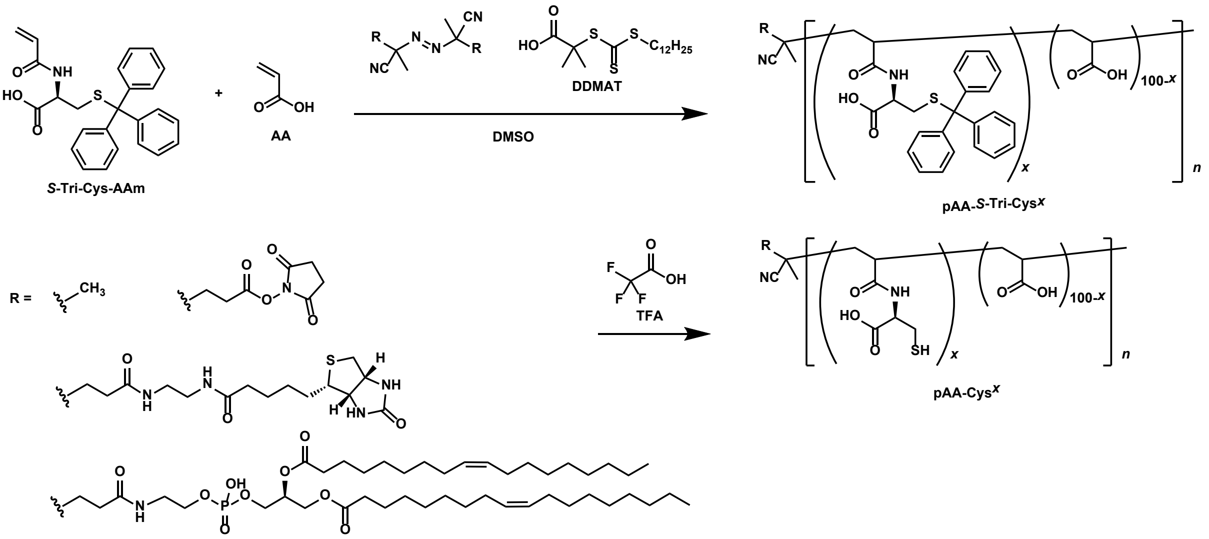

Supplementary Fig. 13. Synthetic scheme for the end-functionalized pAA–Cys5.

Synthesis and Characterization of pAA–Cys5–biotin.

Synthesis of Biotin-initiator (ACVA-biotin)

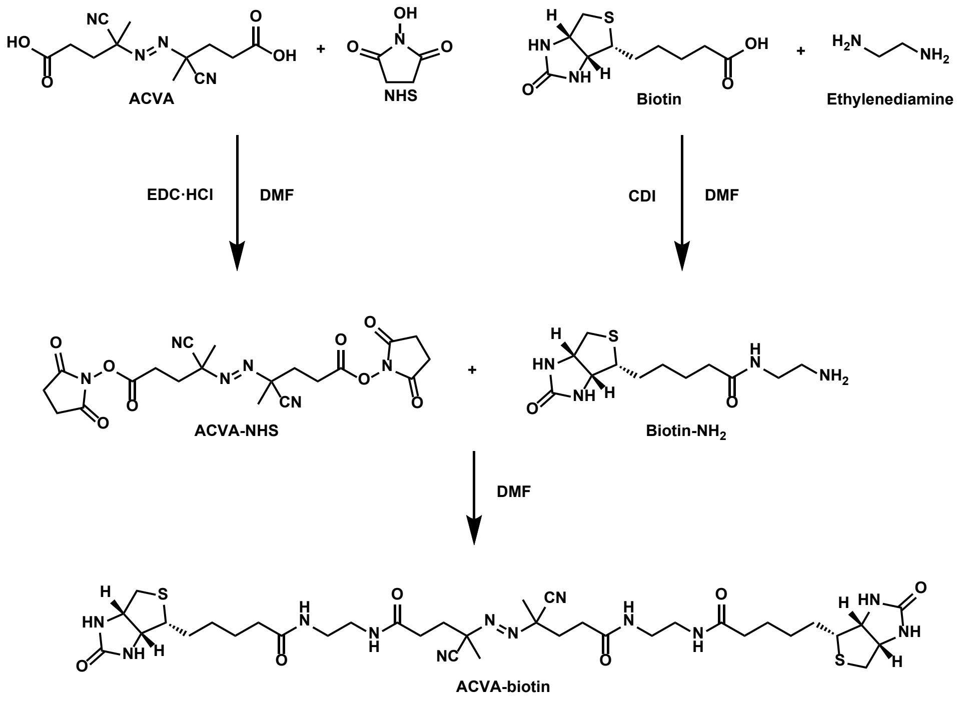

Supplementary Fig. 14. Synthetic scheme for ACVA-biotin.

Preparation of ACVA-NHS: 4,4′-Azobis(4-cyanovaleric acid)-*N*-succinimidyl ester (ACVA-NHS) was prepared according to the procedure by Wang et al.[5] with a slight modification.

ACVA (0.506 g, 1.8 mmol) and NHS (0.557 g, 4.8 mmol) were dissolved in 10 mL of dry DMF (molecular sieves 4A) and cooled in an ice bath. EDC·HCl (0.860 g, 4.5 mmol) was dissolved in dry DMF (25 mL) and then added dropwise at 0 °C over 1 h. After stirring for 1 h on ice, the reaction mixture was warmed to room temperature and allowed to react for 36 h. The solution was then poured into water (350 mL). The resultant precipitate was collected with filtration, washed with water, and dried under reduced pressure to obtain ACVA-NHS as colorless powder (0.667 g, 1.4 mmol, 78%).

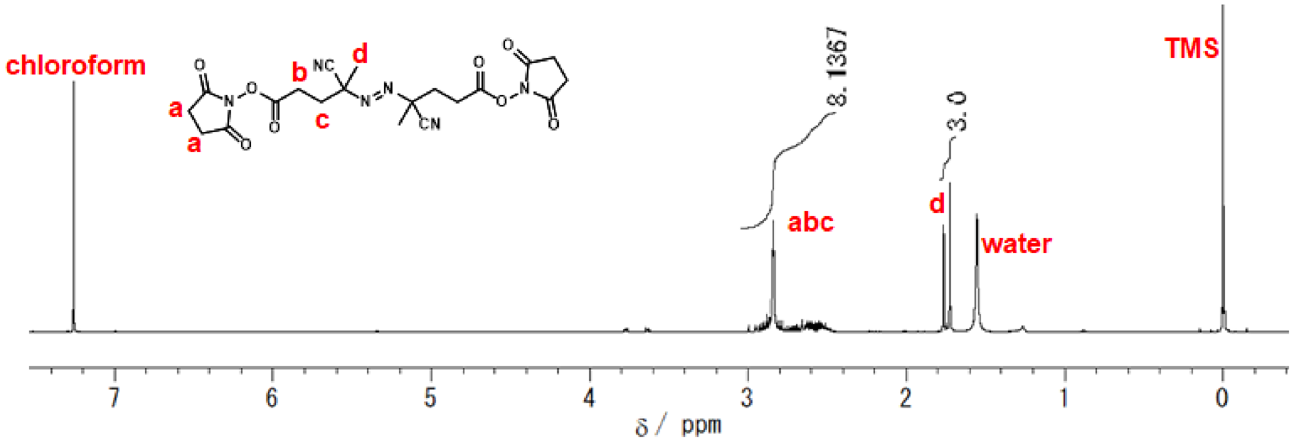

Supplementary Fig. 15. ^{1}H NMR spectrum for ACVA-NHS (400 MHz, $CDCl_3$, 30 °C).

Preparation of Biotin-NH_2: Biotin-NH_2 was prepared according to the procedure by Ramu et al.[6] with a slight modification. (+)-Biotin (1.00 g. 4.1 mmol) was dissolved in dry DMF (13 mL) upon heating (60 °C, 30 min). After cooling to 50°C, CDI (0.799 g, 4.9 mmol) was added. The solution turned into a thick suspension after 5−10 min. This suspension was added to a solution of ethylenediamine (5.0 mL, 75 mmol) in dry DMF (7 mL) at room temperature. The reaction mixture was evaporated to ca. 20 mL to remove excess ethylenediamine and then poured into diethyl ether (200 mL). The resultant precipitate was collected with filtration,

washed with diethyl ether, and dried under reduced pressure at room temperature to obtain Biotin-NH_2 as colorless powder (1.02 g, 3.6 mmol, 87%).

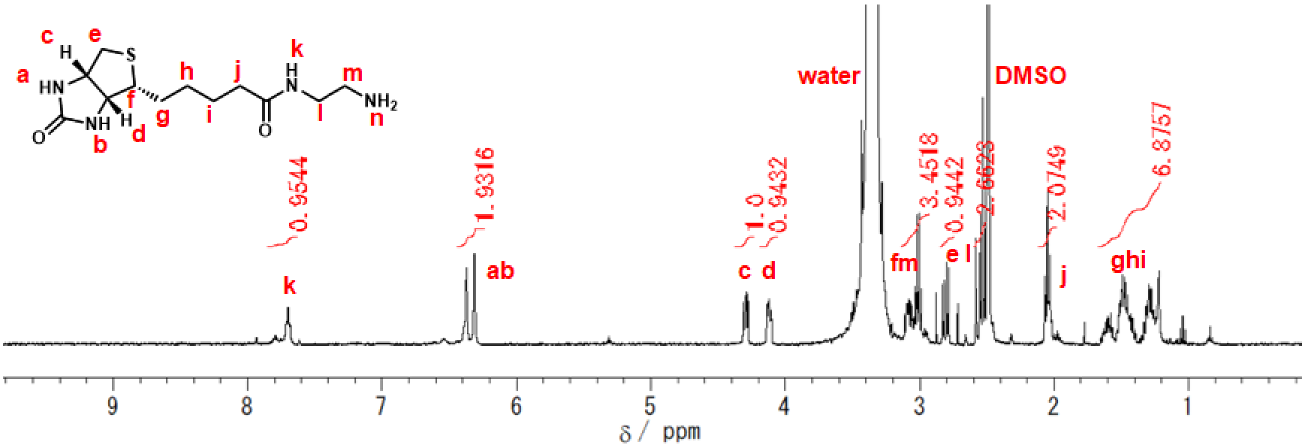

Supplementary Fig. 16. ^{1}H NMR spectrum of Biotin-NH_2 (400 MHz, DMSO-d_6, 30°C).

Synthesis of ACVA-biotin: ACVA-NHS (0.238 g, 0.50 mmol) and Biotin-NH_2 (0.358 g, 1.3 mmol) were dissolved in dry DMF (12 mL). After stirring for 48 h at room temperature, the reaction mixture was poured into diethyl ether (120 mL). The resultant precipitate was collected with filtration, washed with diethyl ether, and dried under reduced pressure at room temperature to obtain ACVA-biotin as a pale-yellow powder (401 mg, 0.49 mmol, 98%).

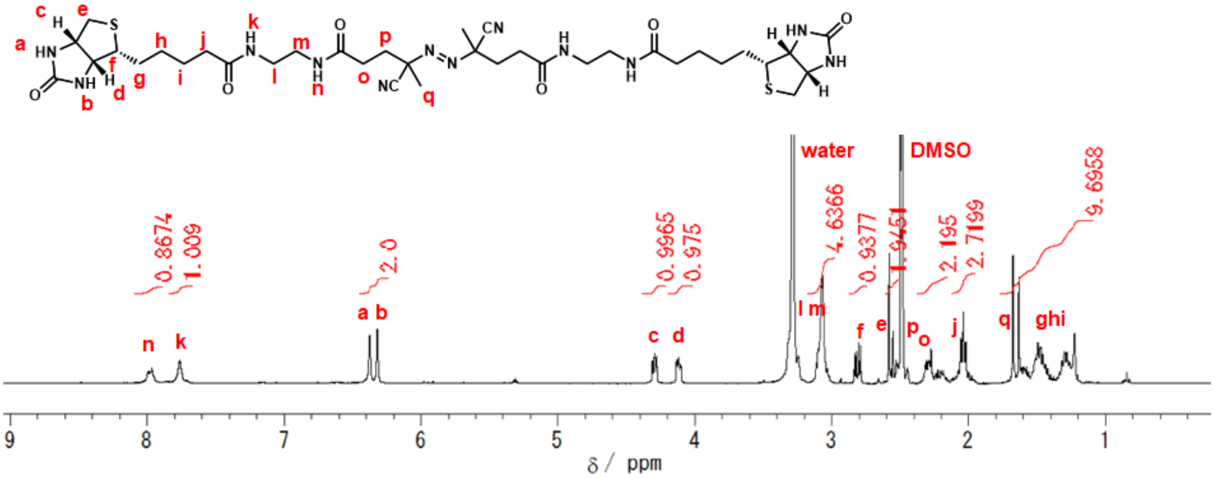

Supplementary Fig. 17. ^{1}H NMR spectrum for ACVA-biotin (400 MHz, DMSO-d_6, 30°C).

Synthesis of pAA–Cys5–biotin

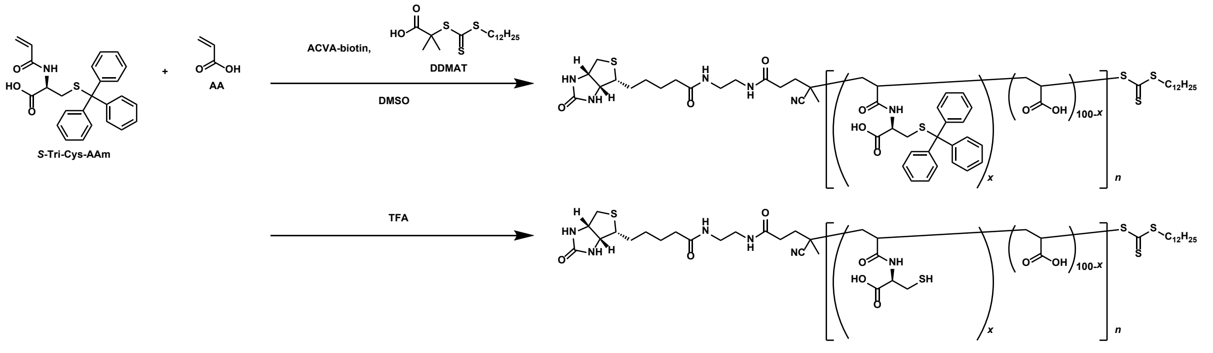

Supplementary Fig. 18. Synthetic scheme for pAA–Cys5–biotin.

pAA–Cys5–biotin was synthesized by RAFT radical polymerization of *S*-Tri-Cys-AAm and AA using ACVA-biotin and DDMAT as an initiator and a chain transfer agent, respectively, followed by deprotection of trityl group with trifluoroacetic acid (TFA). Briefly, *S*-Tri-Cys-AAm (0.05 mmol), AA (0.95 mmol), ACVA-biotin (0.01 mmol), and DDMAT (0.01 mmol) were dissolved in DMSO (1 mL) dried with molecular sieves 4A. The solution was purged with nitrogen gas for 1 h, sealed, and heated in an oil bath at 70 °C overnight. The solution was poured into acetone (10 mL) with stirring. The resultant viscous concentrated phase was collected with centrifugation (3,500 rpm, 5 min). After removing the supernatant by decantation, TFA (1 mL) was added and stirred for overnight. The solution was poured into diethyl ether (10 mL). The resultant solid precipitate was washed with diethyl ether (10 mL) three times, and dried under reduced pressure at room temperature. The weight average molecular weight and polydispersity index (M_w and M_w/M_n, respectively) of pAA–Cys5–biotin were determined by GPC (10 mM Tris-HCl buffer (pH 7.4) + 100 mM NaCl) to be 1.7×10^4 and 2.3, respectively.

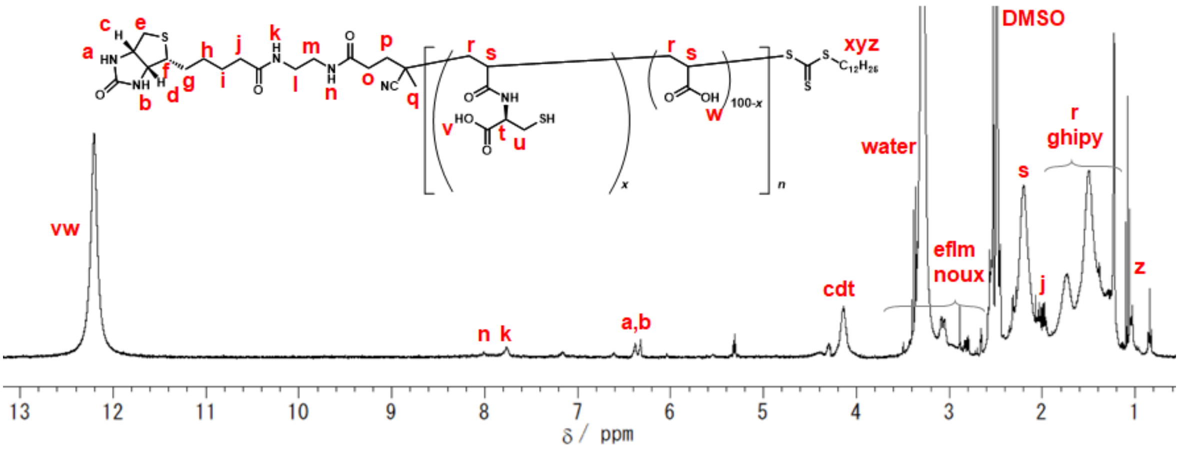

Supplementary Fig. 19. ^{1}H NMR spectrum for pAA–Cys5–biotin (400 MHz, DMSO-d_6, 30°C).

Functionalization of Silica Microparticles with pAA–Cys5.

(a) For the column-based microreactor (Figure 4), we functionalized the surface of silica microparticles in three steps. First, a lipid bilayer was deposited on 10 µm-large silica microparticles (300-10 SIL, VDS Optilab) by incubating small unilamellar vesicles of 1,2-dioleoyl-sn-glycero-3-phosphphatidylcholine (DOPC) doped with 2 mol% of biotin-DOPE for 60 min (total lipid concentration ≈ 1.3 mM).[7] Second, the membrane-coated particles were incubated with neutravidin solution (0.6 µM) for 60 min. Finally, the particles were incubated with the solution of pAA–Cys5-biotin (0.5 µM) for 60 min. After each step, the particles were washed three times with PBS buffer.

(b) For the integrative water treatment system (Figure 5), the surface of the silica particle was functionalized with the monolayer of octadecyltrimethoxysilane.[8] The particles with a diameter of 1.2 µm ((Tokuyama, SS-15)) were incubated with the mixture of DOPC and pAA–Cys5-DOPE (98/2 by mol/mol) dissolved in water/isopropanol for 30 min, then the solvent was step-wisely exchanged to the aqueous buffer following our previous account.[9]

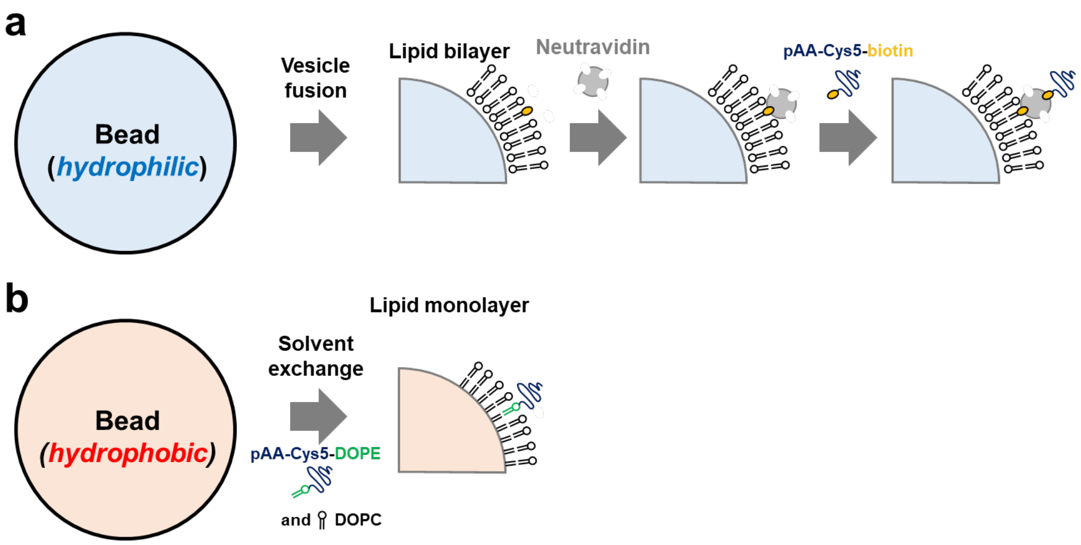

Supplementary Fig. 20. Preparation procedures for (a) pAA–Cys5-biotin immobilized onto lipid bilayer on hydrophilic beads and (b) pAA–Cys5–DOPE immobilized onto lipid monolayer on hydrophobic beads.

Synthesis and Characterization of Fluor–pAA–Cys5.

Fluor–pAA–Cys5–biotin was prepared as reported previously.[7]

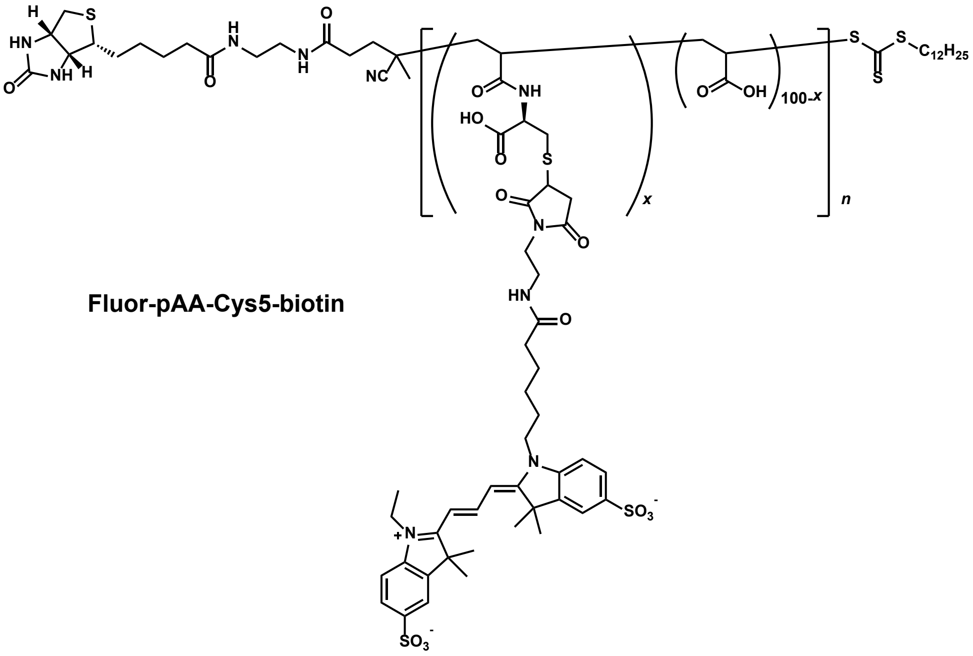

Supplementary Fig. 21. Chemical structure of Fluor–pAA–Cys5–biotin.

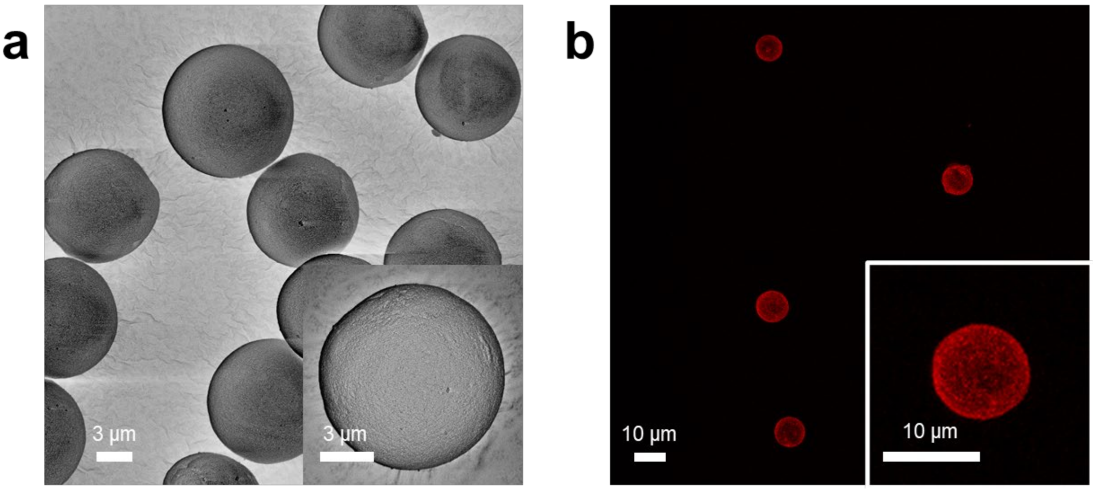

Supplementary Fig. 22. (a) SEM images of silica microparticles (diameter: 10 μm). (b) Fluorescence micrographs of silica microparticles coated with fluorescently labeled pAA–Cys5–biotin (Fluor–pAA–Cys5–biotin, Supplementary Fig. 21).

Synthesis and Characterization of pAA–Cys5–DOPE.

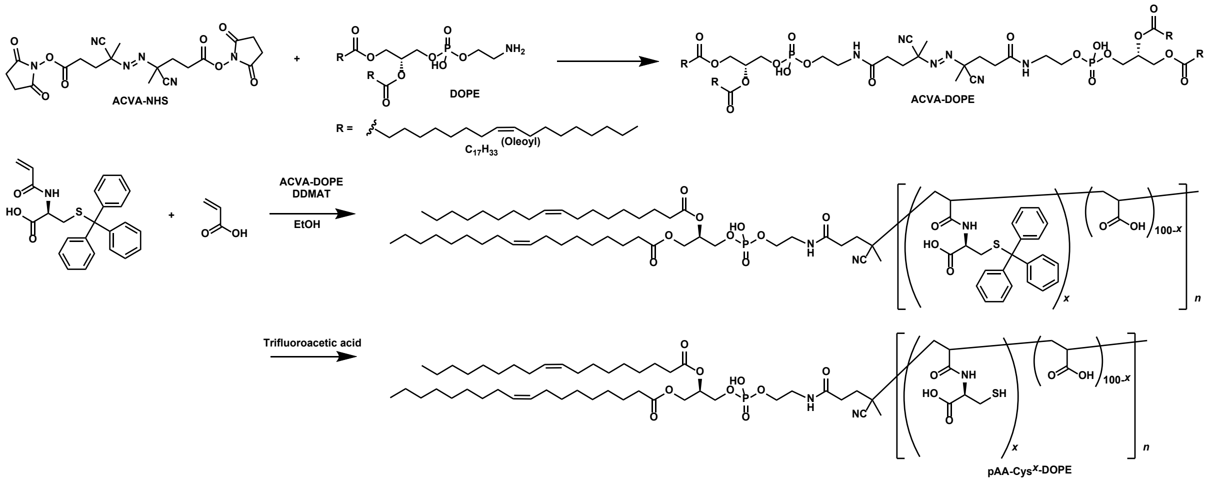

Supplementary Fig. 23. Synthetic scheme for pAA–Cys5–DOPE.

Preparation of ACVA-DOPE

ACVA-NHS (0.252 g, 0.53 mmol), DOPE (0.939 g, 1.3 mmol), and Et_3N (0.18 µL, 1.3 mmol) were dissolved in 50 mL of DCM dried with molecular sieves 4A. Volatile chemicals were evaporated after stirring the solution for 3 days at room temperature. Hexane (10 mL) was added to the residue and allowed to stir for 2 days, followed by filtration with a 0.45 µm poly(tetrafluoroethylene) (PTFE) membrane filter. After evaporation of the solvent, ACVA-DOPE was obtained as a colorless oil.

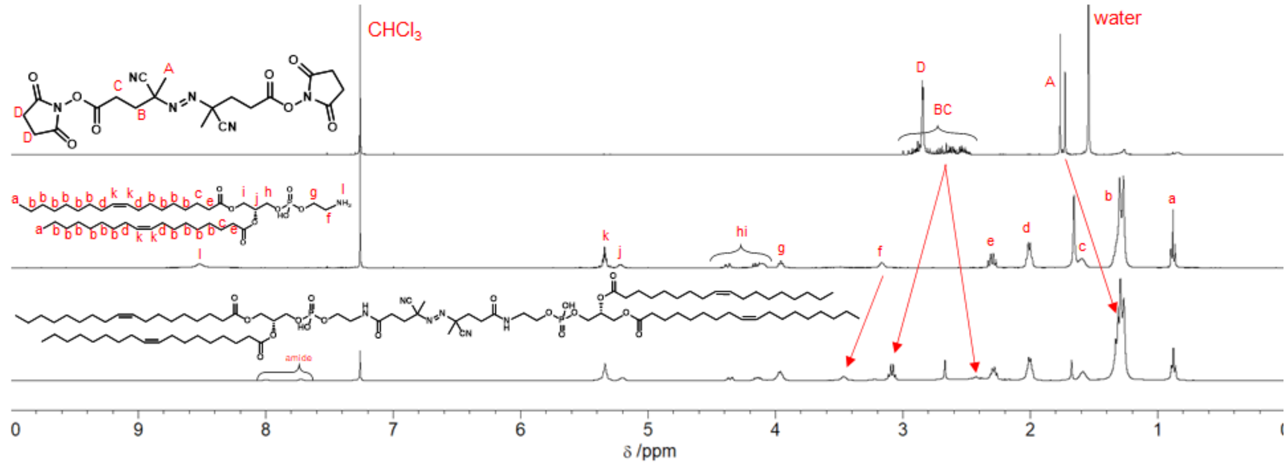

Supplementary Fig. 24. ^{1}H NMR spectra of ACVA-NHS, DOPE, and ACVA-DOPE (400 MHz, $CDCl_3$, 30°C).

Preparation of pAA–Cys5–DOPE

pAA–Cys5–DOPE was synthesized through copolymerization of *S*-trityl-cysteine acrylamide (*S*-Tri-Cys-AAm) and acrylic acid (AA) using ACVA-DOPE and 2-(dodecylthiocarbonothioylthio)-2-methylpropionic acid (DDMAT) as an initiator and a chain transfer agent, respectively, followed by deprotection of trityl group with trifluoroacetic acid (TFA). Briefly, *S*-Tri-Cys-AAm (0.05 mmol), AA (0.95 mmol), ACVA-DOPE (0.01 mmol), and DDMAT (0.01 mmol) were dissolved in 1 mL of ethanol dried with molecular sieves 4A. The solution was purged with nitrogen gas for 1 h, sealed, and heated in an oil bath at 70 °C overnight. After cooling down to room temperature, the solution was poured into diethyl ether (10 mL) with stirring. The resultant precipitate was collected with centrifugation (3,500 rpm, 5 min.). After removing the supernatant by decantation, trifluoroacetic acid (TFA) (1 mL) was added and stirred for 1 h at room temperature. The solution was poured into diethyl ether (10 mL). The resultant precipitate was washed with diethyl ether (10 mL × 2) and dried under reduced pressure at room temperature. Successful polymerization and deprotection were confirmed by ^{1}H NMR measurement. The M_w and M_w/M_n values of pAA–Cys5–DOPE were determined by GPC (10 mM Tris-HCl buffer (pH 7.4) + 100 mM NaCl) to be 7.1 × 10^3 and 1.9, respectively.

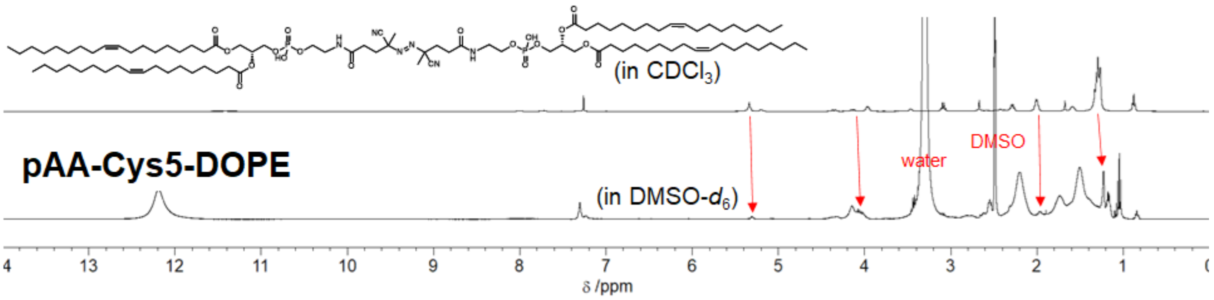

Supplementary Fig. 25. ^{1}H NMR spectra of ACVA-DOPE (400 MHz, $CDCl_3$, 30°C) and pAA–Cys5–DOPE (400 MHz, DMSO-d_6, 30 °C).

Synthesis and Characterization of Chitosan–*g*–pAA–Cys5.

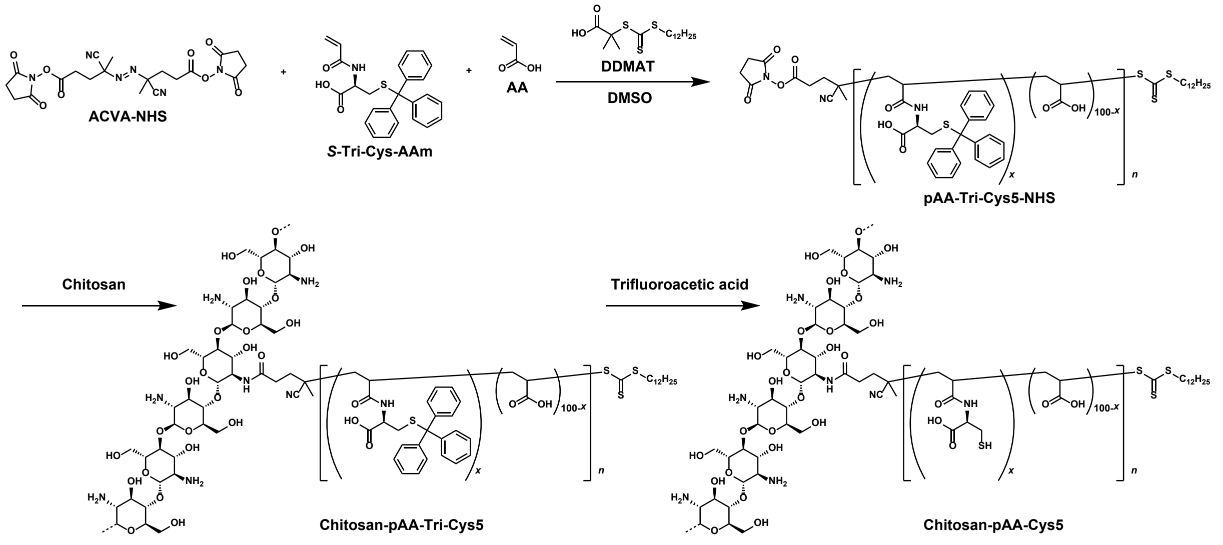

Supplementary Fig. 26. Synthetic scheme for Chitosan–*g*–pAA–Cys5.

Chitosan–pAA–Cys5 was synthesized through grafting pAA–Cys5–NHS onto the amino group of chitosan. Briefly, *S*-Tri-Cys-AAm (0.25 mmol), AA (4.75 mmol), ACVA-NHS (0.05 mmol), and DDMAT (0.05 mmol) were dissolved in 5 mL of DMSO dried with molecular sieves 4A. The solution was purged with nitrogen gas for 1 h, sealed, and heated in an oil bath at 65 °C overnight to obtain pAA–Tri-Cys-NHS solution. Chitosan (medium molecular weight, M_w 1.9-3.1×10^5 g mol^{-1}) (0.212 g) was dissolved in 1 w/v% acetic acid (10 mL). The pAA-Tri-Cys-NHS solution was mixed with the chitosan solution and stirred at room temperature overnight. The mixture was poured into a 10-fold volume of acetone, washed with acetone twice, and the resulting precipitate was recovered by centrifugation (3,500 rpm, 5 min). After discarding the supernatant, the Chitosan-pAA-Tri-Cys5 was obtained after drying under reduced pressure. To the Chitosan-pAA-Tri-Cys5 was added trifluoroacetic acid (10 mL) and stirred at room temperature for 5 h. The Chitosan–pAA–Cys5 was recovered after reprecipitation in a 10-fold volume of diethyl ether, washing with diethyl ether twice, and drying under reduced pressure as a white solid (yield: 0.490 g).

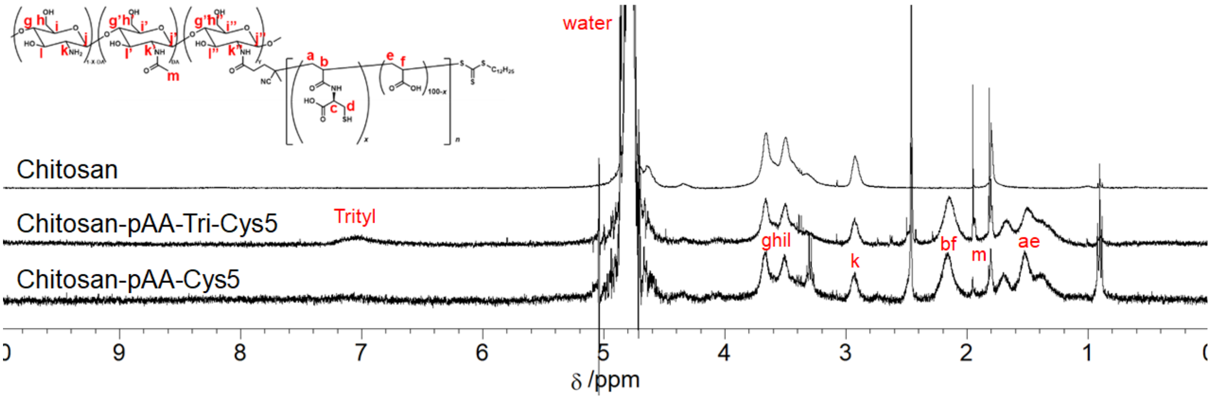

Supplementary Fig. 27. ^{1}H NMR spectra of Chitosan (upper), Chitosan–pAA–Tri-Cys5 (middle), and Chitosan–pAA–Cys5 (400 MHz, 2 v/v% DCl in D_2O, 25°C).

Fabrication of integrative water purification system.

The integrative water purification system is based on the combination of two components; hydrophobized silica particles functionalized with the monolayer of octadecyltrimethoxysilane (Supplementary Fig. 20) and a cellulose membrane coated with chitosan functionalized with *N*-succinimidyl (NHS)-terminated pAA–Cys5 (chitosan–*g*–pAA–Cys5, Fig. 5b). A layer of Chitosan–*g*–pAA–Cys5 was deposited on a cellulose membrane (Φ = 47 mm, Advantec) by the filteration of 5 mg/mL dispersion of Chitosan–*g*–pAA–Cys5 in Milli-Q through the membrane using a KG-47 suction system (Advantec). These membrane and beads were packaged into the Amicon cell (Supplementary Fig. 28).

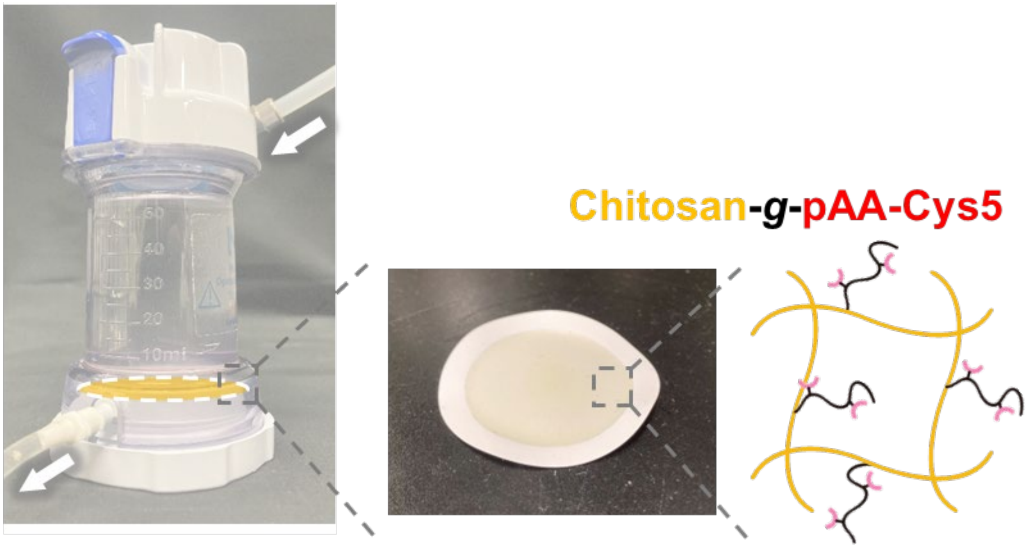

Supplementary Fig. 28. Deposition of a Chitosan-g-pAA-Cys5 layer on a cellulose film.

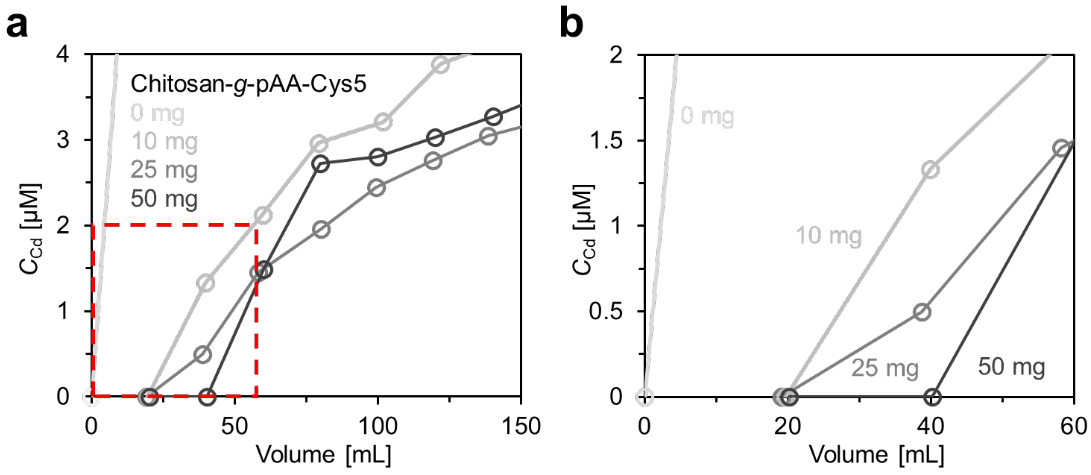

Supplementary Fig. 29. (a) [Cd^{2+}] in the eluent plotted as a function of elution volume using the membrane functionalized with varying amount of chitosan-*g*-pAA–Cys5 (0, 10, 25, and 50 mg) eluted with a buffer containing [Cd^{2+}] = 0.01 mM (10 μM). (b) Magnified view.

Flow-through experiments in the presence of abundant mono- and divalent metal ions in ground water.

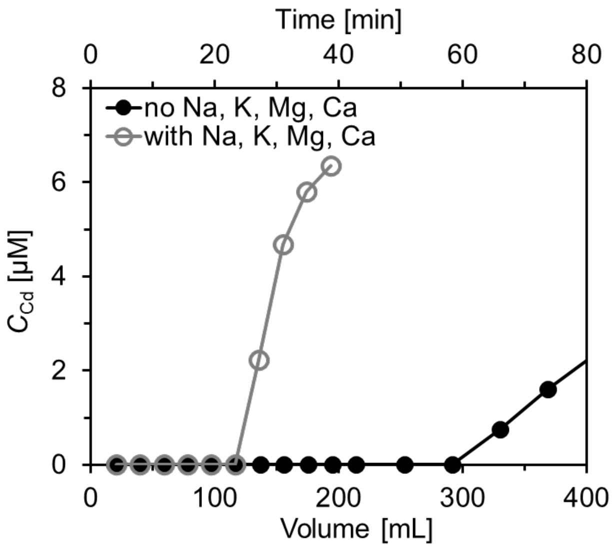

Supplementary Fig. 30. [Cd^{2+}] in the eluent plotted as a function of elution volume using the combination of the membrane and the pAA–Cys5-functionalized microparticles with a buffer containing [Cd^{2+}] = 0.01 mM (10 μM), [Na^{+}] = 1 mM, [K^{+}] = 0.2 mM, [Mg^{2+}] = 0.5 mM, and [Ca^{2+}] = 0.5 mM (gray) and with a buffer containing only [Cd^{2+}] = 0.01 mM (10 μM). (black).

Recovery of pAA–Cys5 by EDTA.

After the saturation (Figure 4), the column was eluted by 10 mM EDTA at a flow rate of 0.01 mL/min for 200 min (total elution volume: 20 mL). The [Cd^{2+}] of the eluent plotted as a function of the fraction number is shown in Supplementary Fig. 31, indicating that the system recovered the Cd^{2+} capture capacity by 83 %.

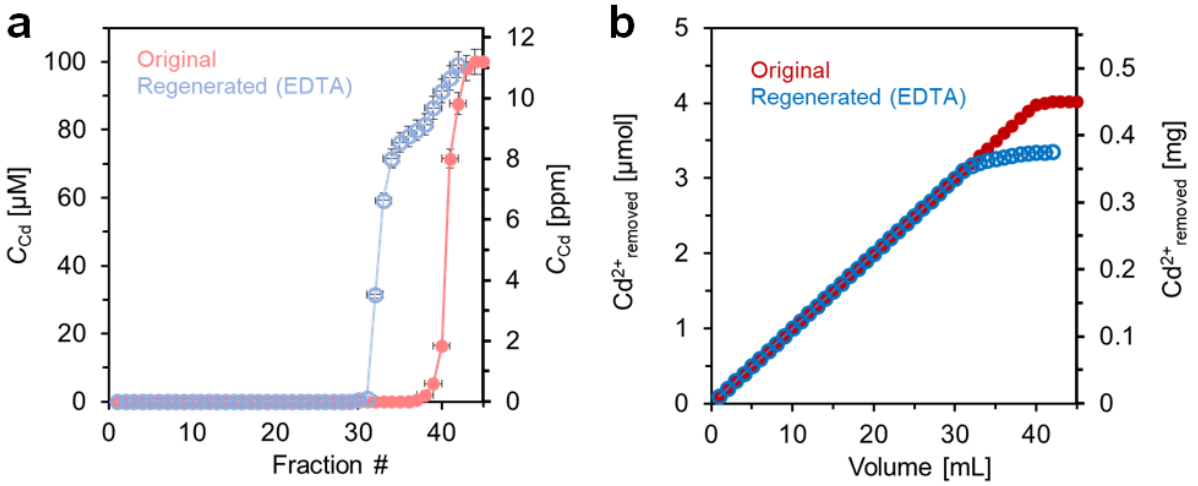

Supplementary Fig. 31. (a) [Cd^{2+}] of the eluent plotted as a function of the fraction number after the treatment with 10 mM EDTA for 200 min. (b) Cumulative amount of Cd^{2+} removed as a function of elution volume.

ITC Measurement for pAA–Cys5 with Hg^{2+}.

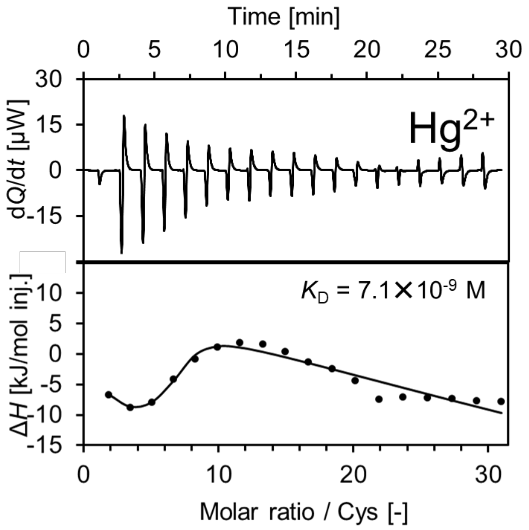

Supplementary Fig. 32. Plots of additional thermal power (d*Q*/d*t*) and enthalpy (Δ*H*) versus the molar ratio of the cysteine side chain by titrating pAA–Cys5 with $HgCl_2$. The best-fit curve for ITC data using two-site model ($HgCl_2$) is shown (solid line).

Supplementary references

1 Podasca, V. E., Buruiana, T., Varganici, C. D. & Buruiana, E. C. Synthesis of non-amphiphilic block copolymer with S-trityl-cysteine and pyrene units by ATRP polymerization: characterization and fluorescence study. *J. Polym. Res.* **24**, 103 (2017).

2 Chekmeneva, E., Prohens, R., Díaz-Cruz, J. M., Ariño, C. & Esteban, M. Thermodynamics of Cd^{2+} and Zn^{2+} binding by the phytochelatin (γ-Glu-Cys)$_4$-Gly and its precursor glutathione. *Anal. Biochem.* **375**, 82-89 (2008).

3 Cheng, Y., Yan, Y.-B. & Liu, J. Spectroscopic characterization of metal bound phytochelatin analogue (Glu–Cys)$_4$–Gly. *J. Inorg. Biochem.* **99**, 1952-1962 (2005).

4 Viswanathan, K., Schofield, M. H., Teraoka, I. & Gross, R. A. Surprising metal binding properties of phytochelatin-like peptides prepared by protease-catalysis. *Green Chem.* **14**, 1020-1029 (2012).

5 Wang, H. *et al.* Synthesis of pyrene-capped polystyrene by free radical polymerization and its application in direct exfoliation of graphite into graphene nanosheets. *J. Polym. Sci., Part A: Polym. Chem.* **53**, 2175-2185 (2015).

6 Ramu, V. *et al.* Two-Photon-Induced CO-Releasing Molecules as Molecular Logic Systems in Solution, Polymers, and Cells. *Chem. Eur. J.* **25**, 8453-8458 (2019).

7 Tutus, M., Kaufmann, S., Weiss, I. M. & Tanaka, M. Functional Coating of Porous Silica Microparticles with Native Biomembranes towards Portable Flow-Through Biochemical Microreactors. *Adv. Funct. Mater.* **22**, 4873-4878 (2012).

8 Hillebrandt, H. & Tanaka, M. Electrochemical Characterization of Self-Assembled Alkylsiloxane Monolayers on Indium−Tin Oxide (ITO) Semiconductor Electrodes. *J. Phys. Chem. B* **105**, 4270-4276 (2001).

9 Yamamoto, A. *et al.* Ion-specific nanoscale compaction of cysteine-modified poly(acrylic acid) brushes revealed by 3D scanning force microscopy with frequency modulation detection. *Nanoscale Adv.* **4**, 5027-5036 (2022).

10 Yamamoto, A. *et al.* Modulation of viscoelasticity and interfacial potential of polyelectrolyte brush by Ion-specific interactions. *Front. Soft Matter* **2**, 959542 (2022).
